# Large-cube 30° × 25° optical coherence tomography in diabetic macular edema

**DOI:** 10.1186/s40942-021-00289-6

**Published:** 2021-03-06

**Authors:** Amir Mahdjoubi, Youcef Bousnina, Fatma-Samia Bendib, Faiza Bensmaine, Wafa Idlefqih, Sadri Chahed, Amina Ghezzaz

**Affiliations:** 1 Department of Ophthalmology, Centre Hospitalier Victor Dupouy, 69 Rue du Lieutenant-Colonel Prudhon, 95100 Argenteuil, France; 2 Department of Ophthalmology, Hôpital Simone Veil, Eaubonne, France; 3 Department of Endocrinology and Diabetology, Centre Hospitalier Victor Dupouy, Argenteuil, France; 4 Department of Ophthalmology, Hôpital Max Forestier, Nanterre, France

**Keywords:** Macular thickness map, Optical coherence tomography, Wide-field imaging, Diabetic macular edema, Diabetic retinopathy, Intravitreal injections

## Abstract

**Background:**

To evaluate the contribution of large-cube 30° × 25° optical coherence tomography (OCT) in the characterization of diabetic macular edema (DME) by assessing its extent and the presence of additional retinal edemas and to evaluate the factors that influenced their occurrence.

**Methods:**

This retrospective study enrolled patients with diabetes who presented with retinal edema detected by horizontal large-cube 30° × 25° (8.7 × 7.3 mm) OCT. Two individualized areas were selected from the thickness map: the area within the 6-mm Early Treatment of Diabetic Retinopathy Study (ETDRS) grid, and that outside the ETDRS grid. Retinal edemas located within the ETDRS grid were designated as “main DME” and those located outside the ETDRS grid were designated as “peripheral retinal edemas.” For each area, OCT features were assessed while the extent of the main DME and the presence of peripheral retinal oedema were analysed in the area outside the ETDRS grid. Finally, part of included eyes was followed by the same protocol, of which a part benefited from intravitreal injections.

**Results:**

Peripheral events were detected outside the ETDRS area in 279 eyes (74.4%) of the 375 eyes of the 218 patients included in this study: an extension of the main DME outside ETDRS grid in 177 eyes (47.2%) and/or the presence of peripheral retinal edemas in 207 eyes (55.2%). The analysis of associations between main DME and peripheral retinal edemas patterns did not find an association for retinal cyst localization (P = 0.42) while a week association was found fort cyst size (Cramer’s V = 0.188, p = 0.028). Nevertheless, a moderate association was found for the presence of microaneurysms (Cramer’s V = 0.247, p < 0.001) and strong association for hard exudates (Cramer’s V = 0.386, p < 0.001), The binary logistic regression analysis retained the following influencing factors of the occurrence of peripheral events: advanced DR stage (Odds ratio OR = 2.19, p = 0.03), diffuse DME (OR = 7.76, p < 0.001) and its location in outer fields (OR = 7.09, p = 0.006). Likewise, the extension of the main DME outside the ETDRS area in was influenced by the same factors in addition to CMT (OR = 0.98, p = 0.004) while the presence of peripheral retinal edema was influenced by the same factors except the outer location of the Main DME. Finally, from the 94 eyes treated by intravitreal injections, extension of the main DME outside the ETDRS grid was detected in 54 eyes (56.44%) at baseline visit and still remained detectable in 37 eyes (39.36%) after treatment initiation.

**Conclusions:**

Large-cube 30° × 25° OCT allowed for more precise assessment of DME extension and better detection of retinal thickening mainly in the advanced stages of diabetic retinopathy with significant DME whether at the baseline visit or during follow-up. The combination of this protocol with a wider ETDRS grid would enhance DME detection and topography.

## Introduction

Optical coherence tomography (OCT) is an essential tool in the diagnosis and management of diabetic macular edema (DME), as it facilitates better detection of DME before retinal thickening becomes visible on slit-lamp biomicroscopy and depicts the quasi-histological details of the retina [1]. Moreover, it has become the gold standard for monitoring treatment response [1–3].

The Early Treatment Diabetic Retinopathy Study (ETDRS) classified DME into clinically significant macular edema (CSME) and non-CSME, based on the location of retinal thickening and/or hard exudates as observed on fundus biomicroscopy or on fundus photography (FP) of macula [4]. For laser treatment, the ETDRS group adopted a grid comprising the radii of three concentric circles. Each circle was divided into four quadrants to form nine fields on FP (a central, inner, and outer fields) [5]. With the introduction of OCT, several classifications based on retinal thickness, distance from the fovea, morphological and quantitative patterns of fluid accumulation, microstructural alterations in the retina, and vitreomacular interface changes have been proposed [6, 7].

The ETDRS grid used in FP was adopted in topography-based classifications, to localize and quantify DME, since the first use of time-domain OCT for the evaluation of DME [8]. Hence, the ETDRS grid was used in DME treatment and assessment its efficiency [3]. Then, the ETDRS grid size was limited to 6 mm and adapted to the maximum OCT field available. However, studies that described the limitations of the acquisition field dimensions of OCT devices reported the lack of information obtained by the ETDRS grid [9, 10].

Recent advances in OCT, including high-speed acquisition, improved image quality, and retinal coverage have resulted in the development of new devices that yield high-resolution images quickly and with larger fields [11]. Moreover, widefield imaging techniques such as ultra-widefield FP, fluorescein angiography, and OCT angiography have improved the understanding and management of diabetic retinopathy (DR) as they provide more details concerning of the retinal periphery [12, 13]. One study analyzed the ability of 55° widefield OCT to detect retinal modifications. The authors found a strong agreement between the conventional and Wide-Field OCT in the detection of morphological features, such as intraretinal fluid, subretinal fluid, hard exudates, micro aneurysms and cotton wool spots. However they neither explored DME-related extension, nor analyzed the features in eccentric locations [14]. To our knowledge, no study has investigated the contribution of a large OCT acquisition field in the analysis DME extent and extramacular retinal edema features in a large cohort.

In this study, we aimed to evaluate the contribution of OCT acquisition with a large 30° × 25° cube in the characterization of DME. First, we evaluated the ability of this protocol to assess the extension of the main DME over the ETDRS grid and the detection of other individualized retinal edemas located outside the classical grid. Subsequently, we aimed to determine whether the retinal edemas located inside and outside the ETDRS grid shared the same patterns by evaluating and comparing the anatomic characteristics of retinal edemas in each area. Furthermore, we evaluated the systemic and local factors that influenced the occurrence of the events outside the ETDRS grids to identify the conditions in which this large acquisition could be more contributive. Finally, we assessed the ability of this protocol to identify residual retinal edemas outside the ETDRS grids after intravitreal treatment.

## Methods

### Patients and clinical data

This retrospective study reviewed the data of 625 eyes of 447 patients who had been examined in the Department of Ophthalmology, Centre Hospitalier Victor Dupouy from January 2017 to April 2019 for DR and had undergone large-cube OCT. The study was approved by the Institutional Review Board of Centre Hospitalier Victor Dupouy and the National Institute for Health Data. All research and measurements were conducted in accordance with the tenets of the Declaration of Helsinki.

The inclusion criteria of this study were as follows: history of diabetes, presence of at least one sign of DR on fundus biomicroscopy, and findings of retinal thickening, hard exudates, and/or retinal cysts on 30° × 25° cube OCT acquisition. Patients with severe opaque media and/or history of other retinal diseases, such as age-related degeneration, retinal vein occlusion, or uveitis were excluded from this study.

We collected from the patients’ medical records the demographic and clinical data: age, gender, history of diabetes and its current treatment, most recent HbA1C levels, and presence/absence of hypertension. The following data from ophthalmological examinations were also selected: eye side, best corrected visual acuity (BCVA), transparency of the optic media, and DR stage based on the International Clinical Diabetic Retinopathy Severity classification.

### OCT acquisition protocol and analysis parameters

We used the Spectralis OCT2 (Heidelberg Engineering, Heidelberg, Germany) for OCT acquisition. This instrument uses an 870-nm central wavelength at an 85-kHz A-scan rate with SP 6.7a software. We used an OCT cube of 30° × 25° (8.7 × 7.3 mm) centered on the fovea, which was formed by 121 horizontal B-scans. The interval between the B-scans was 60 µm and the lateral resolution was 5.64 µm/pixel; the axial resolution was 3.87 µm/pixel, and the frame rate was 10 per B-scan.

**Fig. 1:**
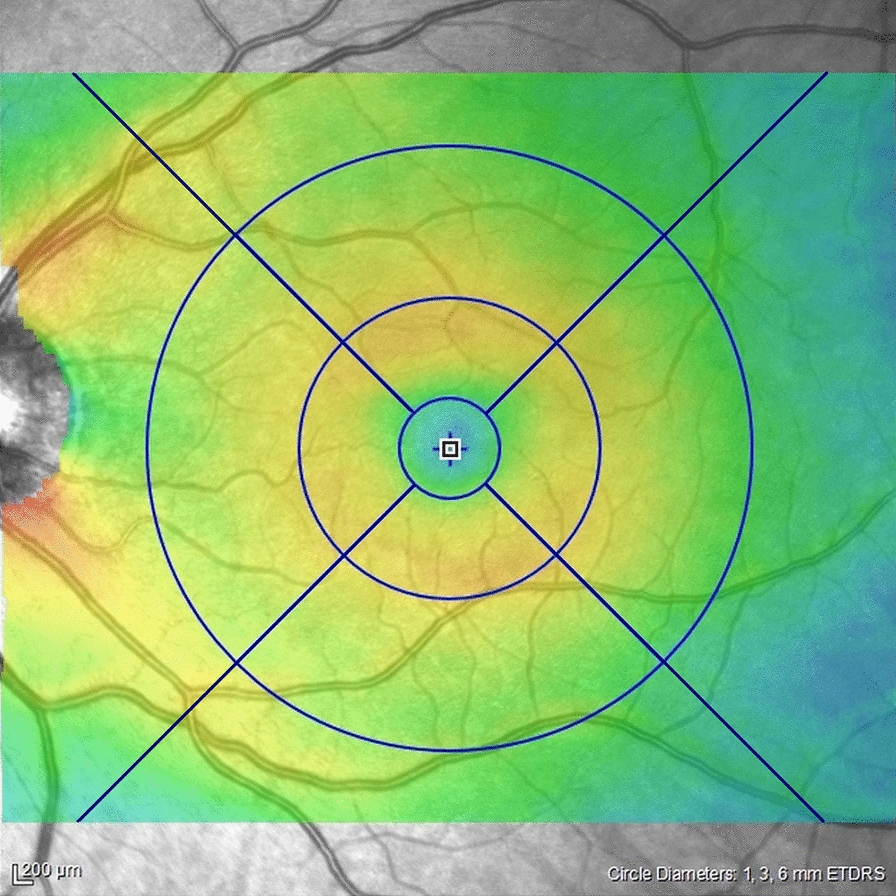
Thickness map of the 30° × 25° OCT cube: The ETDRS grid was centered on the macula and divided by 3 concentric circles (0.5, 3, and 6 mm) into three 3 regions: central, inner, and outer regions. The inner and outer regions were divided into 4 fields to finally form nine fields (central, inferior inner, nasal inner, superior inner, temporal inner, nasal outer, superior outer, temporal outer, and inferior outer). The area outside the ETDRS grid was divided into four fields (inferior, nasal, superior, and temporal) consistent with ETDRS field subdivisions

We generated the thickness map using OCT software that displayed the 6-mm classic ETDRS grid from the acquired OCT images. The ETDRS grid consists of three concentric circles that form the central, inner, and outer regions. The inner and outer regions were divided into four fields to further form nine fields. Thus, we divided the enface thickness map into two areas: the “ETDRS area” and the “outside ETDRS area.” The latter included the retina part located outside the 6-mm circle till the acquisition limits (7.3 mm in height and 8.7 mm in length). This region was also divided into four areas (Fig. 1).

Retinal edema was defined as retinal thickening and/or presence of hard exudates and/or presence of retinal cysts. Depending on the localization, we used the terms “main DME” to designate retinal edema with the highest point located within the ETDRS area and “peripheral retinal edema” to designate retinal edema with the highest point located outside the ETDRS area.

We collected the following data concerning the main DME from the ETDRS area: central macular thickness (CMT), DME surface expansion, greatest retinal thickness and its field localization, size and retinal localization of cysts (if present), and the presence of hard exudates and/or microaneurysms around the retinal thickening area. Cysts were defined as hyporeflective cavities in the form of cystoid spaces located in the retina. Depending on their highest vertical size, the cysts were graded as [6]: small (< 200 µm), intermediate (200–400 µm), and large cysts (> 400 µm). Moreover, cysts were classified according to the retinal layer location in the outer retina (from the outer limiting membrane to the outer plexiform layer), intermediate retina (from the outer to the inner plexiform layer), and/or inner retina (from the inner plexiform layer to the inner limiting membrane). Hard exudates were identified as a homogenous hyperreflective spot located in the outer retina, and microaneurysms were detected as the occurrence of the “ring sign” with hyperreflective circles with a hyporeflective center. Finally, we classified the main DME according to the surface expansion into two classes based on the number of ETDRS fields involved [15]: Focal DME covering three or less thickened ETDRS fields and Diffuse DME covering more than three thickened ETDRS fields.

We observed the presence of “peripheral events” outside the ETDRS area which were defined as the presence of an eventual extension of the main DME outside the ETDRS grid and/or the presence of peripheral retinal edemas. Subsequently, we counted the number of peripheral retinal edemas per eye and collected the following data for each lesion: field localization, highest retinal thickness, size and retinal localization of cysts, presence of hard exudates and/or microaneurysms within the retinal edema, and eventual extension of the peripheral retinal edema into the ETDRS grid.

Finally, these features were also collected from the follow-up visits when they were available for the treated eyes using the same acquisition protocol. Thus, the data were analyzed at the baseline and at the first visit after treatment initiation: 3 weeks after five consecutive monthly intravitreal injections of aflibercept or twelve weeks after one intravitreal injections of Dexamethasone implant (Ozuredex). From these data, we assessed the variation of CMT and highest retinal thickness as well as the presence of residual peripheral event, such as the extension of the main DME and/or peripheral retinal edemas or the appearance of a new one.

The OCT images were acquired and collected by the first (A M) et second reader (A G) and were separately analysed by both of them (both with several years of experience in retinal diseases). After reader agreement analyses, grading discrepancies were resolved by a reader consensus grading of both readers for qualitative features and by calculating the mean for quantitative features.

### Statistics

Statistical analyses were performed using SPSS^®^ version 25 (IBM Corp., Armonk, NY, USA). Descriptive statistics are expressed as the means ± standard deviations for continuous variables and as frequencies and percentages for categorical variables. Cohen’s κ coefficient was employed to quantify the inter-grader agreement for qualitative variables and intra-class correlation coefficient (ICC) for quantitative variables. To assess the relationship between the patterns of the main DME and those of peripheral retinal edemas and to determine factors influencing peripheral events, Pearson’s correlation coefficients were used for quantitative variables, the analysis of variance (ANOVA) were performed for quantitative and qualitative variables with post hoc Bonferroni analysis for multiples testing, and the Chi-squared test was performed for qualitative variables. Cramer’s V was used to assess the strength of association for each Chi square result, with < 0.20 as weak, 0.20 ≤ × < 0.30 as moderate, and ≥ 0.30 as strong [16]. Binary logistic regression was used to confirm the results. All statistical tests were performed at a significance level of α = 0.05 and 95% confidence interval. A p value < 0.05 was considered statistically significant for all analyses.

## Results

### Demographic and clinical data

In total, 375 eyes (192 right eyes; 183 left eyes) of 218 patients (127 men, 91 women) presenting with retinal edema detected using this OCT protocol were included in this study. The mean age was 60.62 ± 13.02 years, while the mean duration of diabetes was 16.75 ± 9.34 years. The mean HbA1C level was 8.99% ± 2.2%. Chronic blood high pressure was observed in 160 patients (77.3%). The demographic and clinical data are presented in Table 1.

**Table 1:** Demographic and clinical characteristics

Characteristics	
Gender (Men/Woman), No (%)	127 (58.2%)/91 (41.8%)
Side (Right/Left), No (%)	192 (51.2%)/183 (48.8%)
Age (years), Mean ± SD	60.62 ± 13.02
High blood pressure, No (%)	160 (77.3%)
History of diabetes (years), Mean ± SD	16.75 ± 9.34
HbA1C rate (%), Mean ± SD	8.99% ± 2.2%
Treatment	
None	4 (1.9)
Oral	42 (20.3%)
Insulin	25 (12.1%)
Oral + Insulin	136 (65.7%)
Missing	11
Diabetic retinopathy stage, No (%)	
Mild	165 (43.8%)
Moderate	130 (34.9%)
Severe	65 (17%)
Proliferate	15 (4.3%)
BCVA LogMAR, Mean ± SD (Snellen)	0.305 ± 0.38 (20/40)
BCVA ≥ 20/40	251 (66.9%)
BCVA classes (Snellen), No (%)	
20/20	103 (27.5%)
20/40—20/25	148 (39.4%)
20/100—20/40	76 (20.2%)
20/125—20/200	21 (5.7%)
< 20/200	27 (7.2%)

*BCVA* best-corrected visual acuity, *SD* standard deviation, *LogMAR* logarithm of the minimum angle of resolution deviation

### OCT features of the main DME and peripheral retinal edemas and their comparison

**Fig. 2:**
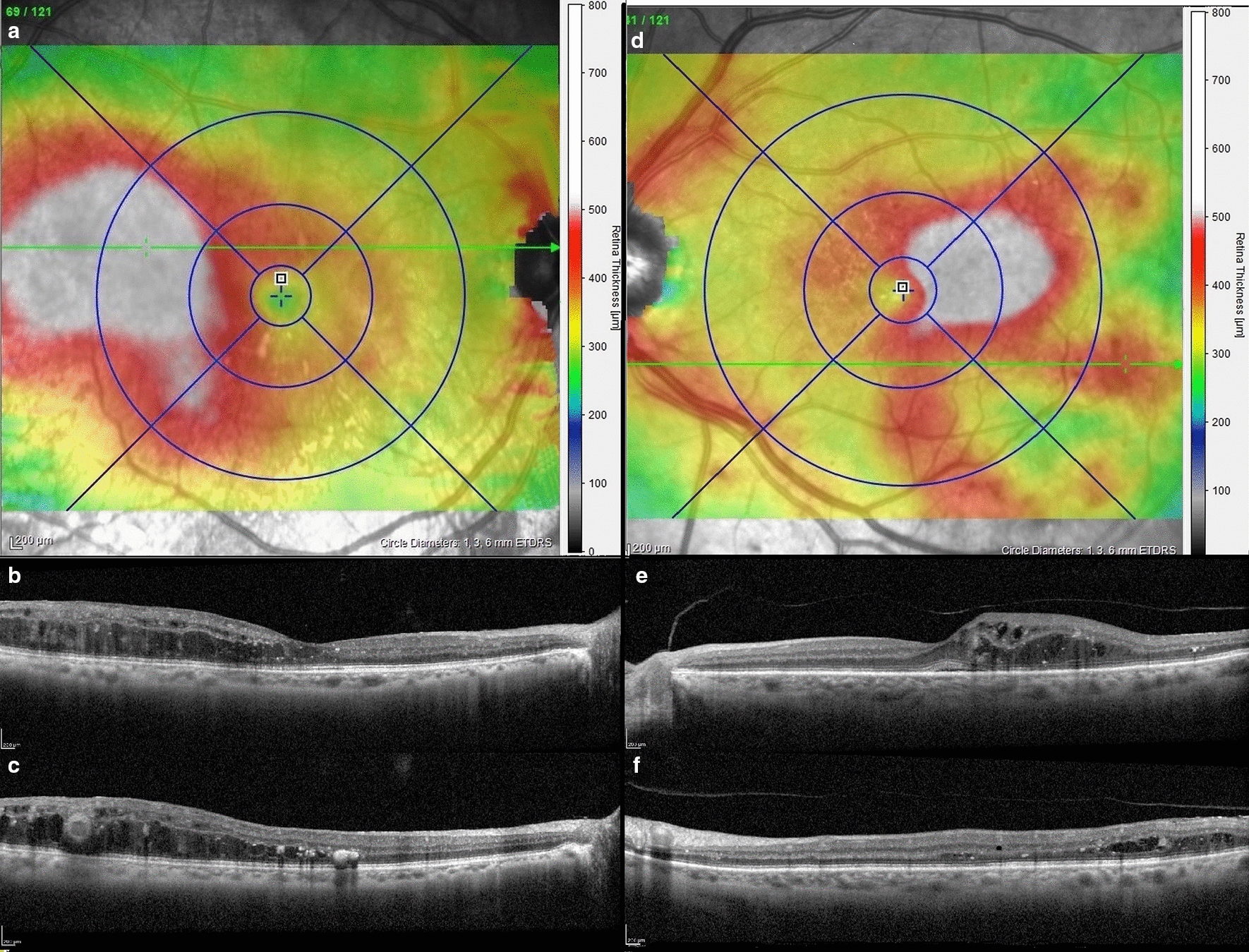
Main DME: **a**–**c **Right eye of a 46-year-old patient with VA of 20/32. The thickness map shows an diffuse main DME involving more than three fields. However, the temporal retinal thickening widely overflowed the ETDRS grid, and the highest point is located in the ETDRS grid. B-scan shows an intermediate cyst in the foveola and small cysts with temporal retinal thickening and hard exudates. We also noted the presence of microaneurysm that are located outside the ETDRS grid and that could be targeted using focal laser. **d**–**f** Left eye of a 69-year-old patient with VA of 20/40. Thickness map shows an diffuse DME associated with three more peripheral retinal edemas, which are detected outside the ETDRS area. They are classified as peripheral retinal edemas as the peak is located outside the ETDRS grid even if they overflow into the ETDRS area. B-scan shows foveolar involvement and the presence of intermediate cysts with hard exudates and microaneurysms. We also noted the presence of intermediate cysts, hard exudates, and microaneurysms in the peripheral retinal edema in the same way as the main DME

**Fig. 3:**
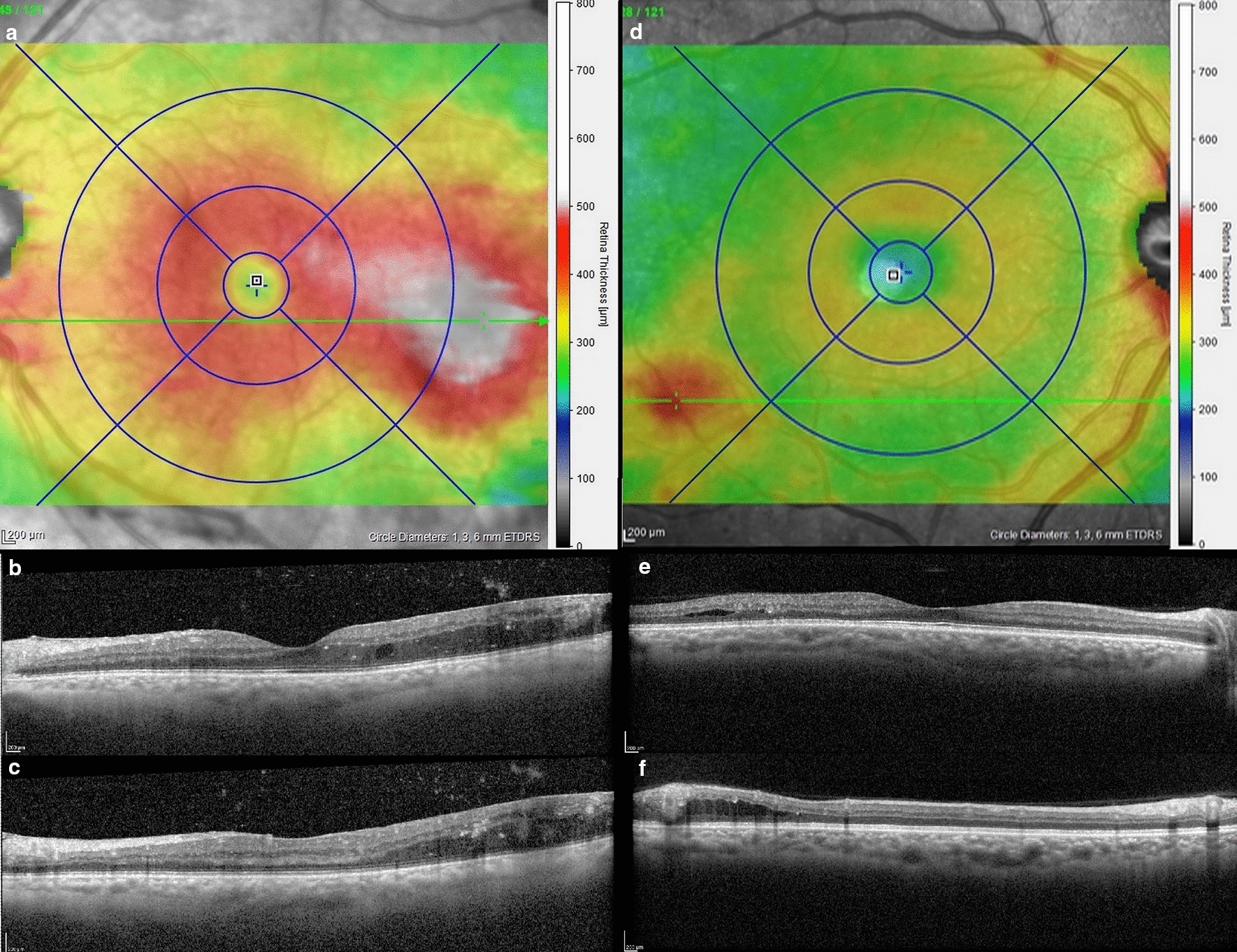
Peripheral retinal edema: **a**–**c** Right eye of a 61-year-old patient with VA of 20/20 with vitreous hemorrhage. Thickness map shows a peripheral retinal edema as the peak is located outside the ETDRS area but that widely overflowed into the ETDRS grid. B-scan displays retinal thickening starting from the peak located peripherally that decrease steadily to reach the foveola. The peripheral peak contains microaneurysms that are probably the origins of the leakage. **d**–**f** Right eye of a 61-year-old patient with VA of 20/20. Thickness map shows no main DME, but an isolated peripheral retinal edema is detected outside the ETDRS area that minimally overflow into the grid. B-scan displays a preserved foveola, and the retinal edema contains a small cyst with hard exudates and microaneurysm

The mean CMT was 304.85 ± 84.16 µm. The ETDRS area analysis indicated that the main DME was found in 339 (90.4%) eyes. It was classified as a focal DME limited to a maximum of three ETDRS fields in 203 eyes (54.1%) and as diffuse DME spanning more than three fields in 136 eyes (36.3%). No main DME was observed within the ETDRS area in 36 eyes (9.6%); only peripheral retinal edemas were detected. The analysis of the area outside the ETDRS grid revealed that peripheral events were observed in 279 eyes (74.4%), including the extension of the main DME outside the ETDRS grid in 177 eyes (47.2%) and/or the presence of a peripheral retinal edema in 207 eyes (55.2%) (Figs. 2 and 3). Of these 207 eyes, only one peripheral retinal edema was found in 129 eyes (34.4%), two peripheral retinal edemas in 58 eyes (15.5%) and three or more peripheral retinal edemas were observed in 20 eyes (5.3%) for a total of 305 peripheral retinal edemas. Inter Observers agreement coefficients were considered as satisfying as they were above 0.85 for all collected parameters. The OCT data of the main DME and peripheral retinal edemas are reported in Table 2.

**Table 2:** OCT features of Main DME compared to those of peripheral retinal edemas

Features	Main DME	Peripheral retinal edemas	P value (coefficient of association)
***Hard exudates, No (%)***	*κ* = *0.92*	*κ* = *0.93*	*** < 0.001***^†^ (0.38)
Absent	45 (13.3%)	17 (5.6%)	
Present	294 (86.7%)	288 (94.4%)	
***Microaneurysms, No (%)***	*κ* = *0.87*	*κ* = *0.86*	*** < 0.001***^†^ (0.24)
Absent	10 (2.9%)	6 (2%)	
Present	329 (97.1%)	299 (98%)	
Cyst Size, No (%)	*κ* = *0.88*	*κ* = *0.89*	***0.027***^†^ (0.18)
Absent	5 (1.5%)	0	
Small	94(27.7%)	225 (73.8%)	
Small + Intermediate	117(34.5%)	69 (22.6%)	
Small + Intermediate + Large	123(36.3%)	11 (3.6%)	
***Cyst location, No (%)***	*κ* = *0.89*	*κ* = *0.90*	0.42^†^ (0.19)
Absent	5 (1.5%)	0	
Outer retina	109 (32.1%)	232 (76.1%)	
Outer + intermediate retina	136 (40.1%)	70 (23%)	
Outer + intermediate + inner retina	66 (19.5	2 (0.6%)	
Intermediate retina	17 (5.0%)	1 (0.3%)	
Intermediate + inner retina	6 (1.8%)	0	
***Location of peak, No (%)***	*κ* = *0.92*	*κ* = *0.93*	
Central	30 (8.8%)		
Inferior Inner	32 (9.4%)		
Nasal Inner	42 (12.4%)		
Superior Inner	41 (12.1%)		
Temporal Inner	76 (22.4%)		
Inferior Outer	18 (5.3%)	60 (19.6%)	0.397^†^ (0.17)
Nasal Outer	13 (3.8%)	28 (9.2%)	
Superior Outer	28 (8.3%)	82 (27.2%)	
Temporal Outer	59 (17.4%)	135 (44%)	
***Highest retinal thickness (µm), Mean ± SD***	*ICC* = *0.95* 409.35 ± 91.27	*ICC* = *0.94* 321.71 µm ± 71.84	*** < 0.001*** (0.42)^‡^

*DME* diabetic macular edema, *ETDRS* early treatment diabetic retinopathy study, *CMT* central macular thickness, *SD* standard deviation

In bold italics: statistically significative (after Bonferroni adjustment)

*κ*: Chohen’s Kappa Coefficient for inter-Observers agreement; ICC: intraclass coefficient for inter-Observers agreement

^†^Chi-Square test (Cramer’s V coefficient)

^‡^Pearson Correlation (Pearson’s r Coefficient)

The analysis of associations between main DME and peripheral retinal edemas patterns did not find an association for cyst localization (P = 0.42) while a week association was found fort cyst size (Cramer’s V = 0.188, p = 0.028). Nevertheless, a moderate association was found for the presence of microaneurysms (Cramer’s V = 0.247, p < 0.001) and strong association for hard exudates (Cramer’s V = 0.386, p < 0.001). Finally, a positive correlation was found between the highest retinal thickness of the main DME and that of the peripheral retinal edema (Pearson’s r = 0.42) (Table 2).

### Factors associated to the extension of Main DME and peripheral retinal edema

To underline the conditions in which this protocol could provide new information, we evaluated the systemic and local factors that are influenced the presence of peripheral event, the overflow of Main DME and the presence of Peripheral retinal edemas. The binary logistic regression analysis retained the following influencing factors of the occurrence of peripheral events: advanced DR stage (Odds ratio OR = 2.19, p = 0.03), diffuse DME (OR = 7.76, p < 0.001) and its location in outer fields (OR = 7.09, p = 0.006). Likewise, the extension of the main DME outside the ETDRS area was influenced by the same factors in addition to CMT (OR = 0.98, p = 0.004) while the presence of peripheral retinal edemas was influenced by the same factors except the outer location of the Main DME (Table 3).

**Table 3:** Univariate analysis and binary logistic regression analysis of factors influencing the peripheral events, extension of Main DME outside ETDRS and the presence of peripheral retinal edema

	Presence of Peripheral events		Extension of Main DME outside ETDRS		Presence of Peripheral retinal edemas	
	Univariate analysis Coeff (p-value) ^† ^	Multivariate analysis Odds ratio [CI] (p value)^‡^	Univariate analysis Coeff (p-value) ^†^	Multivariate analysis Odds ratio [CI] (p value)^‡^	Univariate analysis Coeff (p-value) ^†^	Multivariate analysis Odds ratio [CI] (p value)^‡^
Side	0.01 (0.78)		0/07 (0.14)		0.08 (0.14)	
Age	0.08 (0.08)		0.06 (0.27)		0.04 (0.39)	
Gender	0.06 (0.24)		0.07 (0.17)		0.07 (0.16)	
Diabetes History	0.02 (0.97)		0.07 (0.18)		0.02 (0.68)	
Diabetes Treatment	0.10 (0.28)		0.05 (0.79)		0.07 (0.59)	
High Blood Pressure	0.05 (0.77)		0.02 (0.65)		0.004 (0.94)	
HbA1C	***0.16 (0.007) ***	253.5 [0.001– > 100] (0.54)	***0.18 (0.003) ***	> 100 [0.001–> 100] (0.188)	0.10 (0.06)	
Advanced DR Stage	***0.28 (< 0.001) ***	***2.19 [1.29–3.72] (0.03)***	***0.36 (< 0.001) ***	***1.74 [1.06–2.87] (0.028)***	***0.2 (0.01) ***	***1.42 [1.07–1.88] (0.014)***
CMT	0.05 (0.31)		***0.16 (0.002) ***	***0.98 [0.97–0.99] (0.004)***	***0.13 (0.008) ***	***0.98 [0.97–0.99] (0.005)***
Diffuse DME Surface	***0.39 (< 0.001) ***	***7.76 [2.58–23.32] (< 0.001)***	***0.55 (< 0.001) ***	***23.85 [7.59–74.98] (< 0.001)***	***0.30 (< 0.001) ***	***1.74 [1.00–3.04] (0.027)***
Hard exudates	***0.16 (0.003) ***	2.18 (0.77–6.13) (0.13)	***0.18 (< 0.001) ***	***4.83 [1.27–18.41] (0.021)***	***0.13 (0.01) ***	***2.13 [1.05–4.31] (0.035)***
Microaneurysms	0.007 (0.90)		0.04 (0.44)		0.07 (0.19)	
Large Cysts Size	***0.19 (0.039) ***	1.62 (0.97–2.72) (0.06)	***0.24 (0.001) ***	1.64 [0.90–2.97] (0.10)	0.03 (0.92)	
Outer Cysts location	***0.22 (0.04) ***	0.13 (0.01–1.66) (0.11)	***0.32 (< 0.001) ***	11.57 [0.63–209.50] (0.098)	0.13 (0.29)	
Outer fields location of DME Peak	***0.26 (0.012) ***	***7.09 [1.76–28.57] (0.006)***	***0.28 (0.002) ***	***7.75 [1.65–35.71] (0.009)***	0.25 (0.18)	
Main DME thickness	***0.2 (< 0.001) ***	0.99 [0.98–1.021] (0.10)	***0.34 (< 0.001) ***	1.01 [1.00–1.021] (0.052)	0.01 (0.72)	

*DME* diabetic macular edema, *DR* diabetic retinopathy, *ETDRS* early treatment diabetic retinopathy study, *CMT* central macular thickness, *SD* standard deviation

In bold italics: statistically significative (after Bonferroni adjustment)

^†^Chi-Square test (Cramer’s V coefficient) for qualitative variables and ANOVA for quantitative variables (Point-Biserial Correlation Coefficient)

^‡^Bivariate logistic regression

In parallel, the relationship between the presence peripheral events and BCVA was studied. Even when the latter was stratified according to several systemic and local factors (diabetes treatment, DR stage, DME surface expansion, increased CMT, cyst size and location, presence of microaneurysm, and main DME extending beyond the ETDRS area, patient age, CMT and main DME thickness), the binary regression analysis revealed that a preserved BCVA ≥ 20/40 was more frequent in younger patients [odds ratio OR = 0.88 (0.83–0.93), p < 0.001], and in those in whom the main DMEs did not extend beyond the ETDRS area [OR = 0.88 (0.05–0.98), p = 0.047].

### Evolution of peripheral events during the fellow-up

**Fig. 4:**
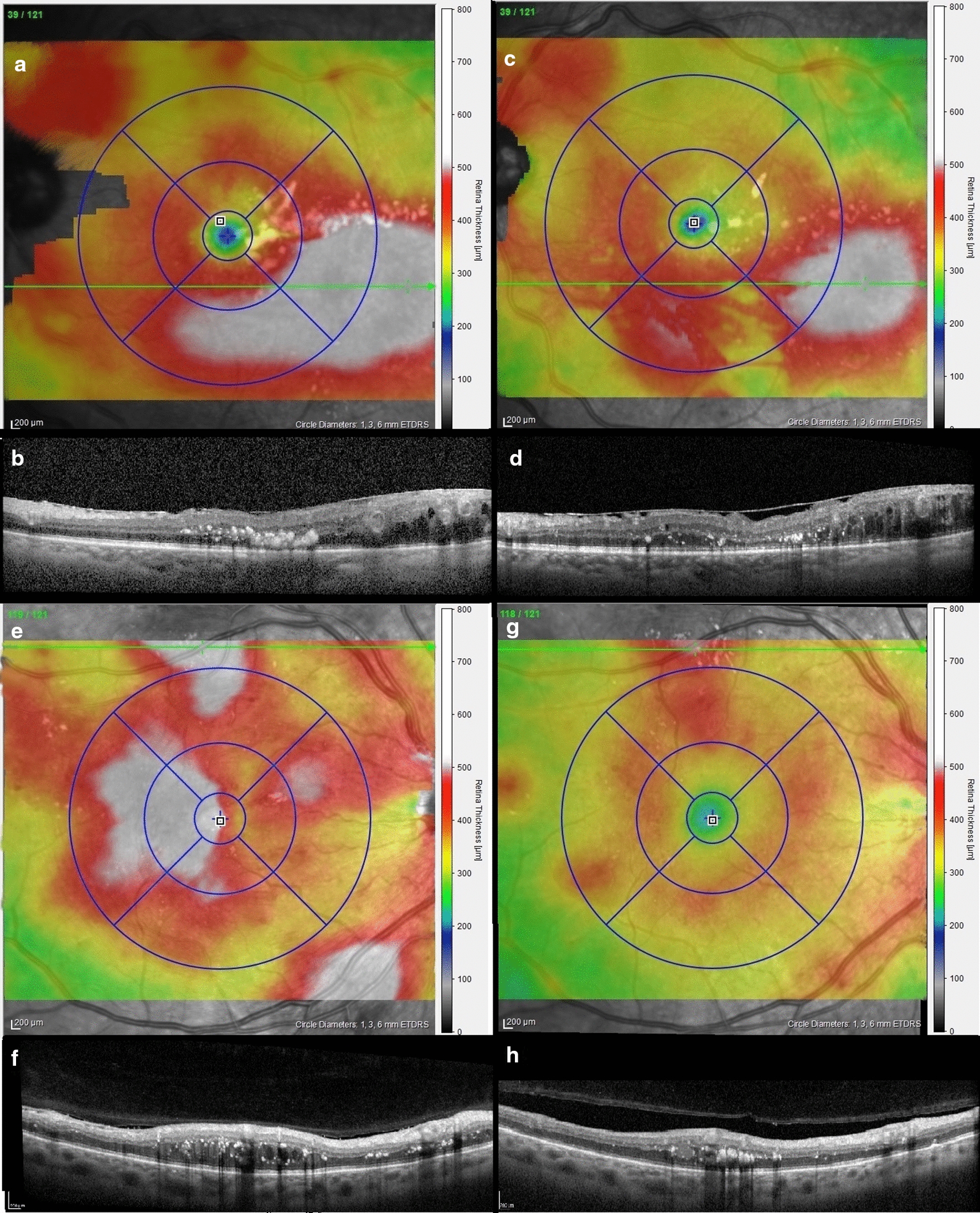
Evolution after intravitreal injections: **a**–**d** Left eye of a 65-year-old woman treated by intravitreal injection of Dexamethasone implant (Ozurdex^®^). Thickness map at baseline visit shows an diffuse DME that widely overflow outside the ETDRS grid in temporal and associated to another peripheral retinal edemas. Twelve weeks after the intravitreal injection, the main DME thickness and surface was reduced but the outside ETDRS part of DME remained thickened with the presence of microaneurysms. The others peripheral retinal oedemas disappeared or regressed. **e**–**h** Right eye of a 43-year-old woman treated by five consecutives by intravitreal injection of aflibercept (eyelea^®^). Thickness map at baseline visit an diffuse DME that slightly overflow outside the ETDRS grid in temporal but associated to two large peripheral retinal edemas in superior and inferior. Three weeks after the fifth injections, the main DME thickness dramatically reduced as well as the inferior peripheral retinal edema. However, the superior peripheral retinal edema remained thickened with the presence of a microaneurysm that could be the source of a DME relapse

A total of 217 eyes that have been followed-up at least one time among them 94 eyes (56.5%) were treated by IVA (59 eyes) or intravitreal Dexamethasone implant (35 eyes). An improvement of CMT was noted in 62 of treated eyes and an improvement of the highest peak of main DME was noted in 75 of the 94 treated eyes (79.78%) (Fig. 4). Interestingly, the extension of the main DME outside the ETDRS grid was detected in 54 eyes (56.44%) at baseline and remained detectable in 37 eyes (39.36%) at the first visit after treatment initiation. It is worth to note that in the non-treated eyes, the extension of the main DME outside the ETDRS was found in 36 eyes at baseline while it was more frequent after the first visit as it was found in 51 eyes. The results comparing the treated and the non-treated patients are presented in Table 4.

**Table 4:** Comparison of the evolution of main DME Extension, CMT and DME highest thickness between treated and non-treated eyes

	Treated eyes (94 eyes)		Non-treated eyes (130)		
	At baseline	After treatment	At baseline	Follow-up visit	
Extension of main DME outside ETDRS	54	37	36	51	P < 0.001*
CMT Above the normal values^†^	52	24	13	20	P < 0.001*
Improvement of CMT^‡^		62		23	P < 0.001*
Improvement of Main DME thickness^‡^		75		45	P < 0.001*

*DME* diabetic macular edema, *ETDRS* early treatment diabetic retinopathy study, *CMT* central macular thickness

In bold italics: statistically significative (after Bonferroni adjustment in univariate analysis)

*Fischer’s Exact Test

^†^CMT > 320 µm for male and > 305 µm for female

^‡^Reduction of retinal thickness more than 10 µm

## Discussion

In this retrospective study, we evaluated the contribution of a large macular OCT cube of 30° × 25° in the characterization of DME by assessing the additional information detected outside the classic ETDRS grid. We found that a large macular acquisition depicts a more precise topography of DME extension and could detect more retinal edemas outside the ETDRS area. The presence of these “peripheral events” was mainly influenced by an advanced stage of DR and meaningful DME. Interestingly, the OCT patterns of the main DME and peripheral retinal edemas were well matched for almost all the collected features.

This protocol yielded supplementary findings from outside the ETDRS area in 74.4% of eyes. Admittedly, data from the ETDRS area are crucial in the diagnosis and management of DME, since the CMT of the 500-μm-radius ETDRS central ring is mostly used, and the central pattern during assessment of disease activity, progression, and treatment response has been examined previously [17, 17] However, there are different manner in which data collected from outside the ETDRS area could contribute to better characterization of the DME and may support its management in the same way of the previous reported information from the non-central-ETDRS fields [9, 18–20].

The fact that the main DME extended beyond the ETDRS grid in 47.2% of eyes in our study indicates that DME extension was more accurately detected in almost half of the cases. Moreover, the extension of the main DME over the ETDRS grid was influenced by several factors such as advanced DR stage, diffuse DME, and CMT. Interestingly, the extension, in turn, was associated with a low BCVA and younger age. These findings are consistent with those reported by Browning et al., who observed a correlation between DME surface extension and baseline CST and BCVA, even if it did not influence BCVA change during the follow-up. However, their study was limited to milder DME cases, using classic ETDRS grid and the authors recommended further evaluation with advanced OCT devices [9]. Similarly, a correlation was found between CMT and the extension of DME to all ETDRS fields in the SAVE protocol [18]. Moreover, the authors revealed that DME extension was less likely to be caused by focal leakage than macular and\or peripheral ischemia. The latter aspect seems to be important, since that the extension beyond the ETDRS area in our study was more frequent in advanced-stage DR when the ischemia was probably more severe. Likewise, Xue et al. reported a positive correlation between the peripheral capillary-drop out on fluorescein angiography and the extension of the DME surface, in line with our results [21]. Importantly, the authors suggested that the presence of extramacular edema should raise the suspicion of peripheral ischemia or neovascularization. Thus, in the advanced stage of DR, the macula would be widely accessible because of capillary disturbance with the increased production of vascular endothelial growth factor (VEGF), which may result in a wider DME. Therefore, a large acquisition would be useful for mapping DME extension, which is a severity marker of both DR and DME in addition to other well-known features.

On the other hand, peripheral retinal edemas located outside the ETDRS grid and disconnected from the main DME were found in 55.2% of eyes. Interestingly, 40.3% of these peripheral retinal edemas extended into the ETDRS grid but were not integrated into the main DME, as the thickest point was located outside the ETDRS area. Moreover, isolated retinal thickening was observed in 9.6% of eyes that did not have any association with the main DME. Thus, this acquisition protocol enhances the sensitivity of OCT for detecting retinal edemas of the posterior pole, as these peripheral lesions could not be thoroughly mapped or even detected by the classical 6X6 mm acquisition protocol. Nevertheless, whether these retinal edemas could be classified as DME extension or just considered as signs of DR remains debatable. For instance, FP was used for the classification of DME before OCT, and the ETDRS grid used was larger than the one adopted for OCT, while the authors explained this explained this issue by highlighting the OCT technology available [22]. Thus, when the original ETDRS grid was used, these peripheral retinal edemas would be included in the grid and. On other hand, a concordance between the main DME and peripheral retinal edemas features was found for almost all the evaluated features. This finding indicates that the peripheral retinal edemas were linked to the main DME and expressed the same characteristics as well as they shared the same severity. Therefore, we think that peripheral retinal edemas could be considered as extensions of the main DME and a wider OCT acquisition will allow to readopt the original ETDRS grid used in FP, which was useful to evaluate laser treatment efficiency.

Practically, wider imaging of DME would be helpful in treatment monitoring, by displaying larger maps of retinal thickness evolution and thus, allowing an eventual detection of residual thickening outside the ETDRS or even identification of recurrence in these areas. In our study, an extension of the main DME beyond the ETDRS grid present at the baseline visit remained detectable in 68.5% of the followed eyes. Consequently, a wider imaging of DME could prompt an earlier intravitreal reinjection or even improve targeting of lesions by laser photocoagulation as an adjunct to injections, especially that many studies have underlined the utility of combined laser and anti-VEGF treatment for BCVA improvement and reduction of the injection number [23, 24].

Moreover, a previous study underlined the benefits of laser treatment in the earlier stages of DME before central involvement. Specifically, focal laser treatment was sufficient for preserving BCVA and OCT thickness in DME without center involvement [10] or even with reduced thickness of the inner and outer fields [25]. Furthermore, Protocol V of DRCR.net showed that focal laser treatment reduces the likelihood of needing aflibercept injections after 2 years in eyes with non-center DME [26]. A better circumscription of an extramacular thickening peak would improve focal laser achievement by more precise laser targeting, especially with the use of navigated laser [27]. Finally, enhanced detection of retinal thickening could be useful in assessment of vision quality since peri-foveolar retinal light sensitivity was correlated with retinal thickening [28] or even in the detection of DR using telemedicine or artificial intelligence [29].

Only a few studies have evaluated the contribution of a large acquisition for DME. One study evaluated the feasibility of a large 45° × 40° field with a prototype and obtained a high-resolution image but did not report any evaluation of DME features [30]. Another study compared the 55° × 25° acquisition to 30° × 25° acquisition by using two different lenses and studied the agreement in detection of the same DME features. Even if more features were identified in the 55° × 25° acquisition, no evaluation of DME extension or detailed retinal edema characteristics were reported [14]. Thus, to our knowledge, this is the first study to evaluate the contribution of a large OCT acquisition field in the analysis of the DME extent and extramacular retinal edema features in a large cohort.

Our study has limitations. First, the study was retrospective and non-interventional, without a control group to compare two distinct acquisitions. However, even when we did not acquire a standard 6 × 6 mm OCT cube, we subdivided the 30° × 25° acquisition into two areas by interposing the ETDRS grid and separately analyzing retinal edemas by zooming into each area. Second, the cube acquired was horizontal rectangular (8.7 × 7.3 mm) and did not extend to the superior and inferior periphery; however, we adopted a horizontal acquisition that covered all posterior poles between the temporal vessels. Third, we used high-resolution parameters, and the B-scan was tightened to precisely study retinal layers, which require longer acquisition time and could be a limiting factor for the routine use of this protocol. However, the acquisition time could be reduced with continuous development of OCT devices. Finally, we used definitions from different DME classifications, which remain to be standardized.

In conclusion, a large OCT cube centered on the macula provided additional information than an acquisition limited to the classic 6 × 6 mm field on DME extension and detected more peripheral retinal edemas, especially in cases of advanced DR with severe DME. Thus, these detected lesions could be diagnosed as DME as they shared the features of the main DME. The adoption of a wider ETDRS grid would account for these lesions and would provide information for DME management especially that the protocol used in this study showed the ability to detect residual rétinal oedema after treatment. A prospective study would assess the contribution of a large OCT cube in the indication and monitoring of DME treatment by different modalities.

## Data Availability

The datasets used and/or analyzed during the current study are available from the corresponding author on reasonable request.
